# Anisotropic Nanocellulose Aerogel Loaded with Modified UiO-66 as Efficient Adsorbent for Heavy Metal Ions Removal

**DOI:** 10.3390/nano10061114

**Published:** 2020-06-05

**Authors:** Jiajia Li, Sicong Tan, Zhaoyang Xu

**Affiliations:** 1 Jiangsu Co-Innovation Center of Efficient Processing and Utilization of Forest Resources, Nanjing Forestry University, Nanjing 210037, China; JiajiaLinjfu@163.com (J.L.); 17768109951@163.com (S.T.); 2 College of Materials Science and Engineering, Nanjing Forestry University, Nanjing 210037, China

**Keywords:** three-dimensional structure, high absorptivity, good mechanical property, directional freeze-drying

## Abstract

Currently, the preparation of outstanding adsorbents has attracted public concern in environmentally friendly and sustainable pollutant redress. Herein, we report a directional freeze-drying method to prepare a strong and reusable adsorbent by introducing metal-organic framework which modified by ethylene diamine tetraacetic acid (named UiO-66-EDTA) into cellulose nanofiber (CNF) aerogel. Compared to traditional aerogels, the fabricated adsorbent showed a good flexibility and reusability by forming a homogeneous three-dimensional structure. By controlling the concentration of a crosslinkable carboxymethyl cellulose (CMC) solution, we produced aerogels with different pore structures and fibrillar, columnar, and lamellar morphologies. The obtained UiO-66-EDTA/CNF/CMC aerogel (U_-EDTA_CCA) showed an excellent adsorption performance for a total of nine types of heavy metal ions, as the removal efficiency could reach 91%. Moreover, the aerogels could retain 88% of their original shape after five cycles. The aerogel may be an appropriate material for the adsorption of heavy metal ions.

## 1. Introduction

Sewage disposal is a global concern subject due to its destructive effect on ecologic sustainability. Wastewater, produced during industrial manufacturing for such goods as textiles, cosmetics, and chemical plants, contains many types of toxic dyes, oils, and heavy metal ions [1,2,3,4]. To address this issue, various technologies have been researched and developed to dispose of heavy metal ions within an aqueous solution; these include membrane filtration [5], chemical coagulation [2], ion exchange [6], and electrochemistry deposition and adsorption [7,8]. Among these technologies, the adsorption was the most attracted method due to its several advantages such as simple preparation, cost-effectiveness, and outstanding removal efficiency [9]. Thus, various adsorbents have been developed to purify heavy metal ions from aqueous solutions [10,11].

Aerogel, as a new generation of 3D network material, possesses many applications such as catalysis [12,13,14], adsorption, and energy storage equipment due to its various superior properties including a high porosity, low cost, and large specific surface area [15,16]. Unlike traditional adsorbents, cellulose nanofiber (CNF) aerogels combine the advantages of both aerogel-type materials and many other superior properties such as rich sources, renewability, biodegradability, and ease of modification [17,18,19]. However, poor mechanical properties often impede the practical application of CNF aerogels as adsorbents, and pure CNFs tend to re-disperse in water, which results in poor reusability. In addition, the adsorption capacity of raw CNF is generally low due to the lack of strong binding sites for specific molecules [20]. In order to overcome these problems, many technologies have been developed to strengthen the mechanical properties of CNF aerogels [21,22,23,24]. Directional freeze-casting has been demonstrated to be a feasible method to prepare materials containing anisotropic pore structures. For example, Hwa et al. reported a sulfur electrode material with a three-dimensionally (3D) aligned structure that enhanced the properties of materials by directional freeze tape casting [25]. Therefore, the directional freeze-casting technology was expected to improve the mechanical properties of CNF aerogels.

Metal–organic frameworks (MOFs) are built from organic ligands and metal nodes, with tunable pore structures and large surface areas, and they have attracted much research attention in different application fields [26,27,28,29]. MOFs have been demonstrated to play a crucial part when purifying wastewater containing heavy metal ions and other different industrial waste dyes [30,31,32]. Because of their natural crystalline property, MOFs are commonly found in powder form at the nano scale, which makes them hard to recycle [33,34]. Therefore, introducing functional MOF materials into template CNF aerogels may not only take advantage of the stable structure of CNF but also maintain the huge surface area and high porosity of MOFs.

In this paper, directional freeze-casting was used to fabricate anisotropic CNF aerogels decorated with modified MOFs (UiO-66-EDTA). The obtained aerogels possessed different pore structures like fibrous, cylindric, or lamellar morphologies and showed both outstanding mechanical properties and a high removal efficiency. Moreover, the U_-EDTA_CCA showed a high stability, as no obvious deformation was observed after several cycles. The prepared aerogels showed a great potential in practical applications for removing heavy metal ions.

## 2. Materials and Methods

### 2.1. Materials

Bleached Douglas fir pulp was bought from Zhongshan NFC Bio-Materials Co., Ltd. (GuangDong, China), and 2,2,6,6-tetramethylpyperidine-1-oxyl (TEMPO, 99.9%), ethylenediamine tetraacetic acid disodium salt (EDTA-2Na), and sodium hypochlorite solution (NaClO) were bought from Alighting Reagent (Shanghai) Co., LTD. (Shanghai, China). Carboxymethyl cellulose (CMC, M.W.250000, DS = 0.7), *N*,*N*-dimethylformamide (DMF), and terephthalic acid (BDC, 99%) were achieved from Sinopharm Chemical Reagent Co. Ltd. Deionized water (DI water, resistivity: 18.25 MΩ cm^−1^) were used to prepare aqueous solution.

### 2.2. Preparation of CNF

The synthesis process of CNF was taken from the literature [35]. Firstly, 10 g of bleached Douglas fir pulp fibers were added into 1000 mL of DI water and smashed with a grinder; then, 0.16 g of TEMPO and 1 g of NaBr were further added into the suspension. Afterwards, NaClO was added dropwise into the suspension with a vigorous stirring, and the pH of the suspension was controlled at approximately 10 using an NaOH solution (0.1 M). Lastly, the suspension of the fiber was passed through a high-pressure homogenizer after chemical pretreatment to obtain the CNF suspension.

### 2.3. Preparation of Modified MOF (UiO-66-EDTA)

MOF (UiO-66) powder was prepared according to the literature [36]. One gram of ZrCl_4_ and 0.713 g of BDC were dissolved in 250 mL of DMF at room temperature, and the mixed solution was stirred vigorously. Simultaneously, acetic acid was dripped into the mixture to control the pH of the solution to faintly acidic. Then, the solution was kept at 120 °C for 24 h. The obtained powder was washed with water and methanol three times before being collected by centrifuge and being dried at 105 °C in the vacuum oven. To obtain the modified UiO-66, 0.1 g of UiO-66 was put into a 50 mL solution containing 3.8 wt% of EDTA. The mixed solution was kept at 60 °C for 24 h without any further operation so that the EDTA molecule could adsorb onto UiO-66. The final product UiO-66-EDTA was washed with DI water at 80 °C for 8 h and then further washed with methanol at 60 °C for 12 h. The UiO-66-EDTA was finally obtained after drying at 60 °C overnight.

### 2.4. Synthesis of Anisotropic U_-EDTA_CCA

First, 0.1 g of modified UiO-66-EDTA powder was added into a 10 mL CNF solution for sonication. Subsequently, CMC with different concentrations of 1, 2, and 4 wt% was added into the suspension (mass ratio of CNF:CMC = 1:1) and vigorously stirred for 1 h. To eliminate impact on the structure and the performance of the U_-EDTA_CCA caused by small bubbles wrapped in the colloidal solution, the prepared mixed solution was put in a vacuum oven at 75 °C for 1 h before being transferred into freezing equipment. The final product was frozen using liquid nitrogen (−196 °C) via directional freeze equipment. The prepared samples were all cylinders with a bottom diameter and a height of 10 mm.

### 2.5. Characterization

Inductively coupled plasma mass spectrometry (ICP-MS, PE, Waltham, USA) was used to measure the concentration of metal ions in the aqueous solution. FE-SEM was used to detect the morphology of the aerogels using a JSM-7600F field emission scanning electron microscope (JEOL, Tokyo, Japan). The FT-IR of the samples were carried out with an FTIR Bruker VERTEX 80 spectrometer (Bruker, MA, USA), and the spectral range of the IR experiment was 4000–600 cm^−1^. XRD was used to determine the crystalline structure of the hybrid aerogels using Cu Kα radiation as a source (Rigaku, Tokyo, Japan). The XPS of the samples was measured by using an AXIS UltraDLD (Shimadzu, Kyoto, Japan) with Al Kα X-ray as the excitation source. The surface area of the MOFs and the modified MOFs were measured by the Brunauer–Emmett–Teller (BET) theory (ASAP 2020 HD 88, Micromeritics, Norcross, USA), and all the samples were pretreated at 105 °C for 10 h to degasify in a vacuum before testing.

### 2.6. Density and Porosity of Hybrid Aerogels

The density (ρ) and porosity (*P*, %) of the U_-EDTA_CCA aerogel was figured by Formulas (1) and (2):

(1) $$\rho =\frac{m}{V},$$

(2) $$P=\frac{V-m/\rho_{c}}{V}\times 100\%,$$

where m represents the mass (g) of the aerogel, *V* represents the volume (cm^3^) of the aerogel, and $\rho_{c}$ is the density of the solid density of each component.

### 2.7. Heavy Metal Ions Adsorption Experiments

In this paper, Cr^3+^, Cu^2+^, Co^2+^, Ni^2+^, Mn^2+^, Zn^2+^, Sn^4+^, Fe^3+^, and Zr^4+^ were chosen as pollution elements and were prepared in an aqueous solution by dissolving them in DI water. The adsorption equilibrium was achieved by pouring 0.004 g of the U_-EDTA_CCA aerogel into 10 mL of water containing heavy metal ions for 5 days. The pH of the aqueous solution containing heavy metals was adjusted by the 0.1 M HCl and 0.1 M NaOH solution to keep it neutral. Afterwards, aerogels adsorbed with metal ions were thoroughly filtered through a membrane to remove impurity from the solution. The concentration of the solution containing heavy metal ions after adsorption was measured by ICP-MS. The adsorption capacity *Q_t_* (mg g^−1^) of the absorbents and the removal percentage *E* (%) were calculated using Equations (3) and (4), respectively:

(3) $$Q_{t}=\frac{\left(C_{0}-C_{t}\right)}{M_{A}}\cdot V_{H},$$

(4) $$E=\frac{\left(C_{0}-C_{e}\right)}{c_{0}}\times 100\%,$$

where *C*_0_ (mg L^−1^) represents the initial concentration, *C_t_* (mg L^−1^) represents the concentration at time *t*, *C_e_* (mg L^−1^) represents the equilibrium concentrations, *M_A_* (g) represents the weight of adsorbent, and *V_H_* (L) represents the volume of heavy metal ion solutions.

The rate constant of adsorption corresponded Equations (5) and (6):

(5) $$k_{1}t=ln\left(\frac{Q_{e}}{Q_{e}-Q_{t}}\right),$$

(6) $$\frac{t}{Q_{t}}=\frac{1}{k_{2}Q_{e}^{2}}+\frac{t}{Q_{e}},$$

where *Q_e_* (mg g^−1^) and *Q_t_* (mg g^−1^) represent the adsorption capacities at equilibrium and the adsorption capacities at time *t* (h), respectively, and *k*_1_ is the rate constant of the pseudo-first-order model. The pseudo-second-order kinetic model is used to fit the variation law of adsorption amount and time, wherein *k*_2_ is the rate constant of the pseudo-second-order kinetic model.

## 3. Results and Discussion

### 3.1. Preparation of U_-EDTA_CCA

**Figure 1:**
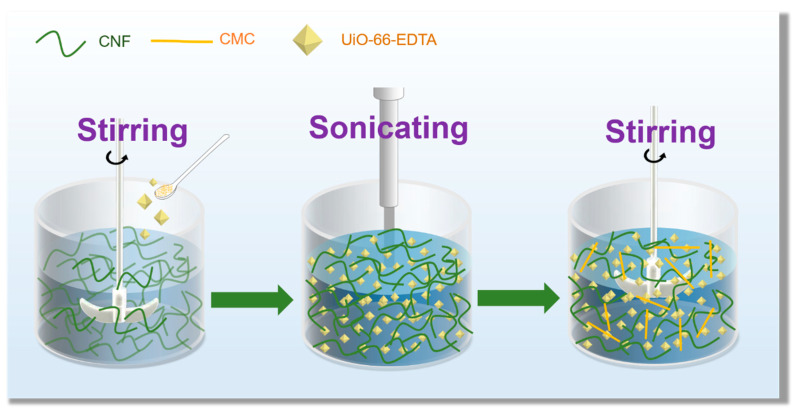
Experimental process of the UiO-66-EDTA-containing a cellulose nanofiber (CNF) suspension.

**Figure 2:**
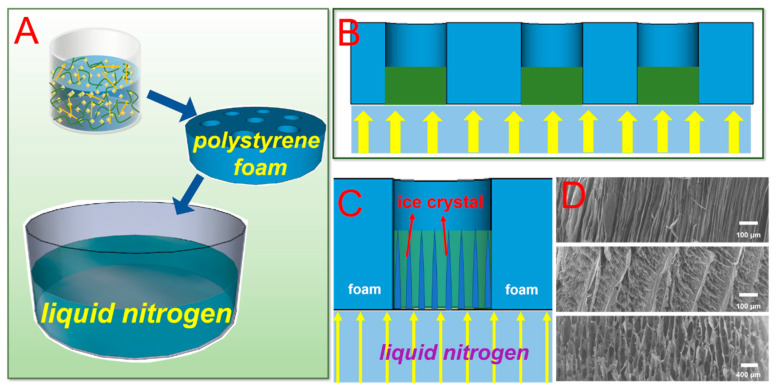
(**A**–**C**) The experimental process of directional freeze-drying. (**D**) Morphology of the U_-EDTA_CCA via directional freeze-drying.

Figure 1 shows the preparation procedure of the U_-EDTA_CCA. The adsorbent was mainly formed by loading functional UiO-66-EDTA into a nanocellulose aerogel network. Figure 2 shows a schematic illustration of directional freeze-drying. Water was rapidly frozen into ice crystals and grown vertically in the horizontal direction via directional freeze-drying so that anisotropic U_-EDTA_CCAs was formed (Figure 2D). By controlling the concentration of CMC, aerogels with fibrous, cylindric, and lamellar structures were produced. In this paper, the anisotropic U_-EDTA_CCAs showed a low density (0.005 g cm^−3^), a high porosity (99%), and good mechanical properties, which greatly improves the application prospect of U_-EDTA_CCAs in the field of heavy metal adsorption.

### 3.2. Surface Morphology Analysis

**Figure 3:**
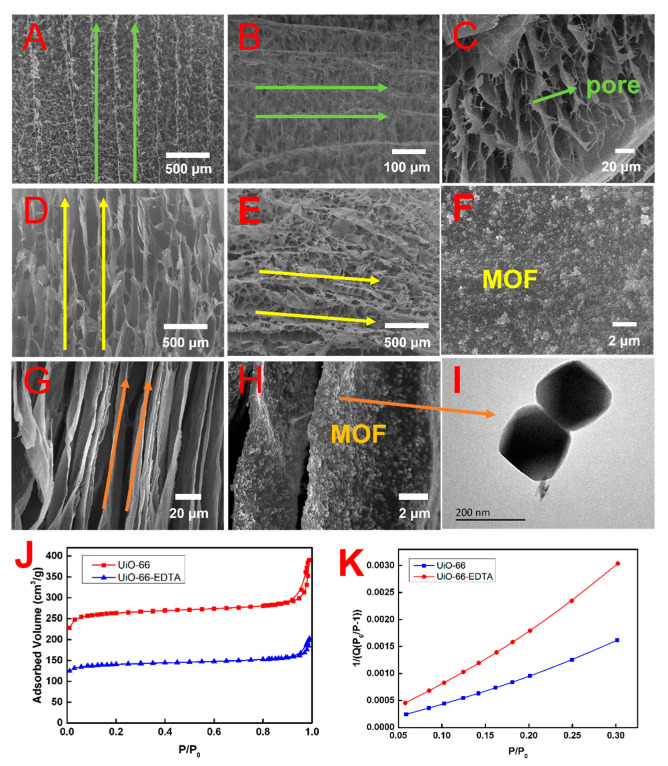
(**A**,**D**,**G**) FE-SEM images of the direction of ice; (**B**,**E**) FE-SEM images of the top view of the U_-EDTA_CCAs; (**C**) pore structure of U_-EDTA_CCAs; (**F**,**H**) FE-SEM images of UiO-66-EDTA particles loaded onto the “walls” of aerogels; (**I**) TEM images of UiO-66-EDTA; (**J**,**K**) N_2_ adsorption/desorption isotherms and BET surface area plots of UiO-66 and UiO-66-EDTA at 77K, respectively.

The surface morphology and alignment degree of U_-EDTA_CCAs were examined by FE-SEM. Figure 3A,D,G shows the direction of the ice-growth, which exhibited three general trends. By increasing the concentration of CMC from 1 to 4 wt%, the morphology of the hybrid aerogel changed from a fibrillar structure to a cylindric structure and finally changed into a lamellar form. Figure 3B,E shows the top view of the U_-EDTA_CCAs. Apparently, there was a visible anisotropic pore structure not only on the vertical side but also on the horizontal side. In Figure 3F,H, it can be observed that the UiO-66-EDTA nanoparticles were loaded on the laminar of the aerogels. In general, the UiO-66-EDTA particles were dispersed in the aerogel pore walls, and no particle agglomeration was observed (Figure 3F,H). It may be concluded that anisotropic pore structure of the aerogels loaded with the UiO-66-EDTA particles were successfully fabricated via directional freeze-casting. This anisotropic pore structure could improve the adsorption efficiency and enhance the mechanical properties of aerogels. As is shown in Figure 3I, the structure of the UiO-66-EDTA was not damaged after modification. Figure 3J,K shows N_2_ adsorption/desorption isotherms and the BET surface area plots of UiO-66 and UiO-66-EDTA. All the samples were pre-treated at 102 °C for 10 h for degasification in a vacuum before testing. The BET surface areas of UiO-66 and UiO-66-EDTA were calculated to be 851 and 582 m^2^ g^−1^. In this paper, we found that the aerogel with a columnar structure performed well in adsorption, mechanical, and reuse, so the following performance tests were all columnar structure aerogels without special instructions.

### 3.3. Chemical Composition

**Figure 4:**
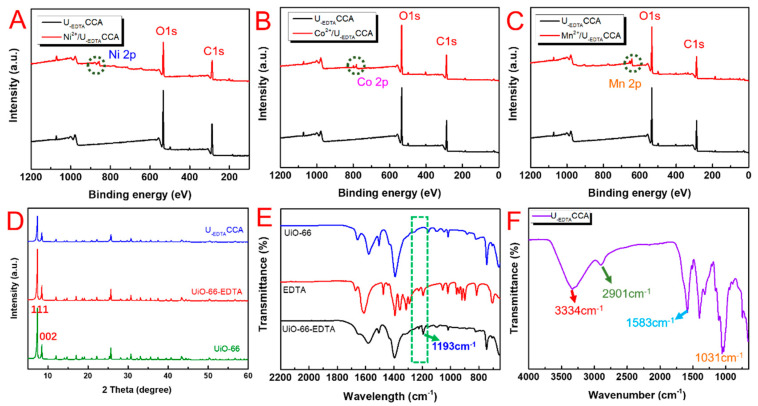
(**A**–**C**) Comparison of the wide-scan XPS spectra of the U_-EDTA_CCA loaded with heavy mental ions; (**D**) XRD patterns of UiO-66, UiO-66-EDTA, and U_-EDTA_CCA; (**E**) FTIR spectra of UiO-66, EDTA, and UiO-66-EDTA; and (**F**) FTIR spectra of U_-EDTA_CCA.

XPS was used to examine the chemical components of the samples. Wide-scan XPS patterns of the samples are shown in Figure 4A–C. Core levels located at 855.3, 783.1, and 643.4 eV were attributed to Ni^2+^, Co^2+^, and Mn^2+^, respectively. To demonstrate that the crystalline form of UiO-66-EDTA was not destroyed by the modification, an XRD test was performed. A comparison of the diffraction peaks is shown in Figure 4D. Two characteristic peaks located at 7.4° and 8.5° corresponded to the (111) and (002) planes of UiO-66, respectively, which demonstrated that the crystallinity of UiO-66 was well-preserved. The high similarity of the samples indicated that UiO-66-EDTA was successfully grown on the hybrid aerogel surfaces. FTIR patterns of the samples are shown in Figure 4E,F. In Figure 4E, the peak of 3334 cm^−1^ was due to -OH stretching vibration. The peaks centered at about 1656–1395 cm^−1^ were due to the bond of the C=O stretching vibration in the carboxylic acid, the bond of C=C stretching vibration in a benzene ring, and the O–C–O stretching vibration in the BDC ligand. The peaks of 2901 and 1031 cm^−1^ were caused by the stretching vibration of C–H and C–O–C, respectively. The wide peak ranged from 1110 to 1164 cm^−1^ might have been caused by the asymmetric tensile vibration of the glycosidic ring. In addition, the FTIR spectra of UiO-66-EDTA showed that the newly appeared peak centered at 1193 cm^−1^ was caused by the C–N stretching band, which indicated that EDTA was successfully grafted onto UiO-66.

### 3.4. Adsorption Performance Analysis

**Figure 5:**
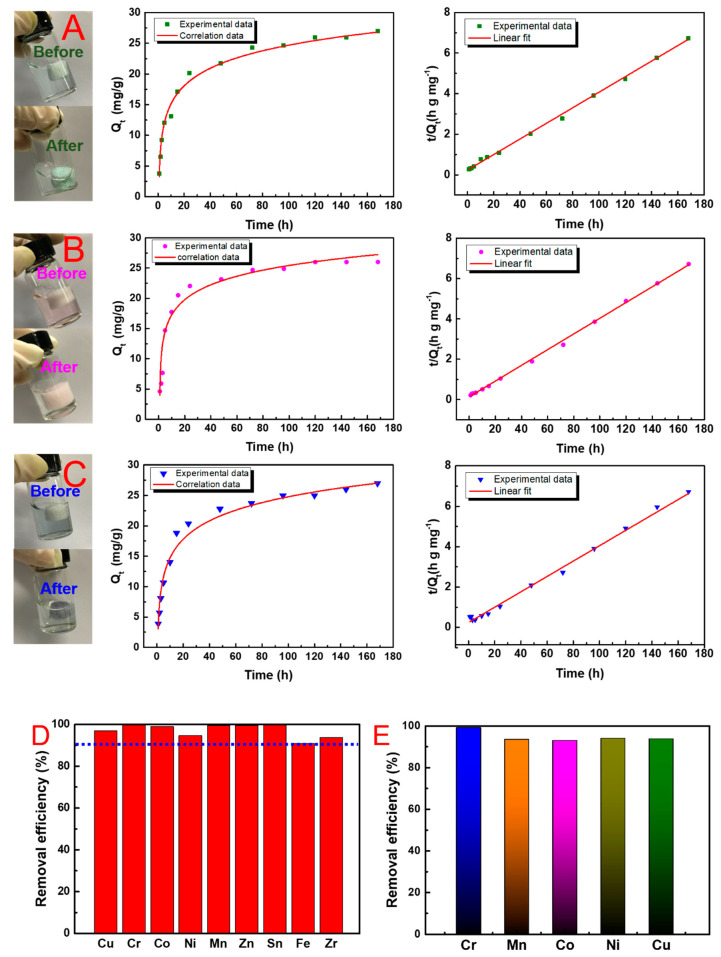
Time-dependent adsorption. (**A**–**C**) The adsorption kinetics of adsorption of Cu^2+^, Co^2+^, and Cr^3+^ via U_-EDTA_CCA. (**D**) The removal efficiency of heavy metal ions for U_-EDTA_CCA. (**E**) The simultaneous removal efficiency for Cr^3+^, Mn^2+^, Co^2+^, Ni^2+^ and Cu^2+^ metal ions in batch adsorption.

In this paper, nine kinds of typical metal elements were selected to prepare the contaminated solution to evaluate the adsorption efficiency of U_-EDTA_CCA. U_-EDTA_CCA was added into a 10 mL aqueous solution containing heavy metal ions with a concentration of 10 mg L^−1^ under the condition of pH = 5. As can be seen in Figure 5A–C, kinetic behavior was studied by testing the U_-EDTA_CCA’s adsorption capacities (Q_t_) at different times. In this paper, the adsorption properties and reusability of U_-EDTA_CCA were much better than those of pure CNF aerogels. The excellent adsorption ability could be attributed to the powerful chelating between target ions and EDTA in hybrid aerogels. Figure 5D presented the removal efficiency of U_-EDTA_CCA for each measured metal ion, and the results showed that the best removal rate of heavy metal ions was 98%. Considering the complex composition of wastewater, the U_-EDTA_CCA was put into a mixed solution containing five kinds of metal ions to test its removal efficiency for mixed metal ions. The result is shown in Figure 5E, and the high removal efficiency (>91%) indicated that U_-EDTA_CCA was a potential material for purifying wastewater containing various metal ions. For comparison, adsorbents prepared by similar materials are listed in Table 1; clearly, U_-EDTA_CCA was the best. Above all, large pores at the micron level and the anisotropy pore structure of the CNF aerogels were beneficial for the fast removal of heavy metal ions and promoted the contact between functional UiO-66-EDTA particles and heavy metal ions. In addition, aerogels after adsorbing were easy to collect compared to pure MOFs in the powder form.

**Figure 6:**
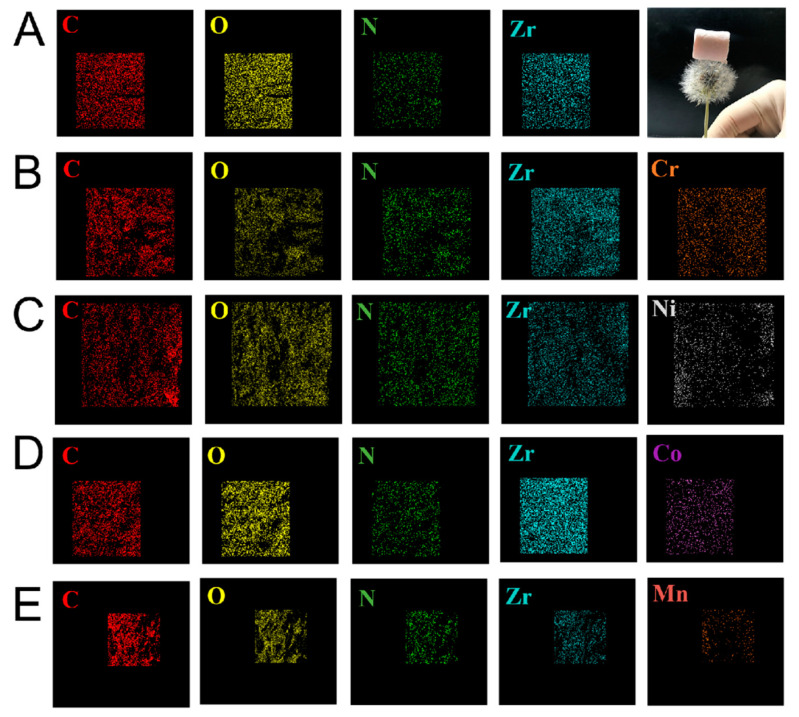
(**A**) Corresponding elemental maps for U_-EDTA_CCA. (**B**–**E**) single-metal system U_-EDTA_CCA loaded with Cr^3+^, Ni^2+^, Co^2+^, and Mn^2+^, respectively.

Heavy metal ion-loaded U_-EDTA_CCA aerogels were further measured via elemental mapping using SEM. In Figure 6A, elemental mapping indicated that zirconium was well-dispersed in U-_EDTA_CCA, which could be used as particular metal-connected sites for the command of metal ions’ dispersion in aerogel adsorbents. Figure 6B–E showed the elemental mappings of specific metal ion-loaded U_-EDTA_CCA. It was shown that Cr^3+^, Ni^2+^, Co^2+^, and Mn^2+^ were uniformly dispersed in the aerogels, thus providing good proof of our hypothesis.

### 3.5. Recycling of Adsorbents

**Figure 7:**
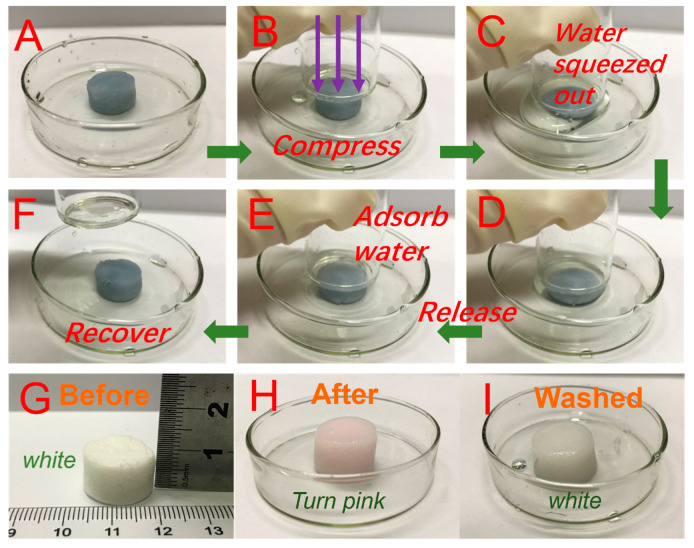
(**A**–**F**) The adsorption process for heavy metal ions of the U_-EDTA_CCA; (**G**) geometric size of the U_-EDTA_CCA; (**H**) U_-EDTA_CCA loaded with Co^2+^; and (**I**) image of the regenerated U_-EDTA_CCA.

**Figure 8:**
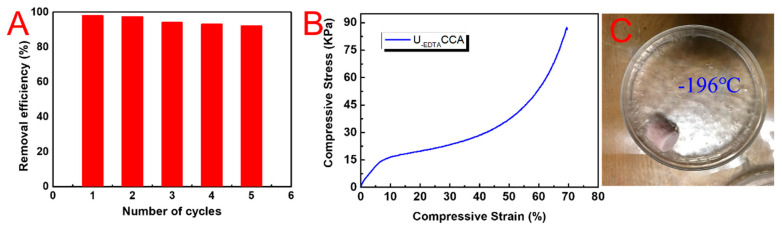
(**A**) Removal efficiency of Cr^3+^ of U_-EDTA_CCA; (**B**) compressive stress–strain curve of U_-EDTA_CCA; and (**C**) the stability of U_-EDTA_CCA at −196 °C.

For practical application, the reusability of the samples was also tested. Figure 7A–F shows the resilience of U_-EDTA_CCA. Obviously, U_-EDTA_CCA loaded with Cr^3+^ could recover its original shape after the mechanical squeezing process without any deformation, which demonstrated its excellent mechanical properties. It is noteworthy that the U_-EDTA_CCA loaded with metal ions could be reused by washing it with a high concentration of the EDTA-2Na solution. Figure 7G–I shows that the color of the aerogel changed from white to pink after being loaded with Co^2+^ ions, and it returned back to the white color after being washed with the EDTA-2Na solution. This phenomenon demonstrated that the prepared aerogels could be easily recycled without destroying their inner structure. The regenerated aerogels were further used as samples in the following metal ion removal experiments. The U_-EDTA_CCA retained over 88% of its adsorption capacity for all the measured metal ions after five cycles (Figure 8A). As shown in Figure 8B, U_-EDTA_CCA could be compressed to 70% of its original shape without mechanical failure and presented 86 KPa at a 70% strain. The U_-EDTA_CCA showed good stability after recycling five times without ab obvious reduction in performance. Furthermore, we found that the aerogels showed a good low temperature resistance. As seen in Figure 8C, the U_-EDTA_CCA in the Petri dish containing liquid nitrogen (−196 °C) could maintain its original shape without damage.

## 4. Conclusions

The novel adsorbent U_-EDTA_CCA was prepared by combining the functional UiO-66-EDTA and CNF into an anisotropic aerogel followed by directional freeze-casting methods. The prepared aerogel exhibited a good absorptivity and outstanding shape stability under extrusion. By controlling the concentration of the CMC solution, aerogels produced three types of pore structures with fibrillar, columnar, or lamellar morphologies. The adsorption experiment for total nine kinds of heavy metal ions showed that the removal efficiency was more than 91%. Apparently, anisotropic pore structure was beneficial for improving the adsorption efficiency. Moreover, the U_-EDTA_CCA showed excellent stability after five cycles without obvious deformation, and the removal efficiency was still over 88%. The U_-EDTA_CCA, with good reusability and high adsorption properties, may have potential applications in the field of removing heavy metals.

## Figures and Tables

**Table 1:** Comparison of the adsorption of metal ions of U-_EDTA_CCA with other adsorbents.

Adsorbents	Qmax (mg g^−1^)								Reference
	Cu^2+^	Cr^3+^	Co^2+^	Ni^2+^	Mn^2+^	Zn^2+^	Fe^3+^	Zr^4+^	
UiO-66-NH2@CA	39								[37]
Am-WS		136							[38]
2C-g-PAN			100	49					[39]
3CMC-g-PAA/MT						286			[40]
4Phos-CNCSL							115		[41]
Carboxymethylated cellulose fiber	17			12					[7]
Activated carbon					172				[42]
U-EDTACCA	104	396	307	120	251	215	115	165	This work
